# Changes in CD4+CD25HIGH T cells and TGFβ1 levels in different stages of adult-onset type 1 diabetes

**DOI:** 10.5937/jomb0-49868

**Published:** 2024-11-16

**Authors:** Tanja Miličić, Aleksandra Jotić, Ivanka Marković, Dušan Popadić, Katarina Lalić, Veljko Uskoković, Ljiljana Lukić, Marija Maćešić, Jelena Stanarčić, Milica Stoiljković, Mina Milovančević, Đurđa Rafailović, Aleksandra Božović, Nina Radisavljević, Nebojša M. Lalić

**Affiliations:** 1 University of Belgrade, Faculty of Medicine, University Clinical Centre of Serbia, Clinic for Endocrinology, Diabetes and Metabolic Diseases, Belgrade; 2 University of Belgrade, Faculty of Medicine, Institute for Medical and Clinical Biochemistry, Belgrade; 3 University of Belgrade, Faculty of Medicine, Institute for Microbiology and Immunology, Belgrade; 4 University of Belgrade, Faculty of Organizational Sciences, Department for Operations Research and Statistics, Belgrade

**Keywords:** CD4+CD25high T cells, first-degree relatives of patients with type 1 diabetes, TGFb, type 1 diabetes, CD4+CD25high T ćelije, TGFb, prvi rođaci pacijenata sa tipom 1 dijabetesa, tip 1 dijabetesa

## Abstract

**Background:**

Previous studies suggested an important role of impairments in T cell subsets in different stages during type 1 diabetes (T1D) development, while data regarding CD25high T cells and transforming growth factor b1 (TGFβ1), both T regulatory associated, remains controversial. We analyzed the level of (a) CD25high T cells (b) TGFβ1 in 17 first-degree relatives of patients with T1D in stage 1 (FDRs1) (GADA+, IA-2+); 34 FDRs in stage 0 (FDRs0) (GADA, IA-2); 24 recent-onset T1D in insulin-requiring state (IRS); 10 patients in clinical remission (CR); 18 healthy, unrelated controls (CTR).

**Methods:**

T cell subsets were characterized by two-color immunofluorescence staining and flow cytometry; TGFβ1 was determined by ELISA, GADA, and IA-2 by RIA.

**Results:**

The percentage of CD25high T cells in FDRs1 was lower than controls, FDRs0, IRS, and CR (p<0.001). Additionally, the cut-off value for CD25high = 1.19%, with a probability of 0.667, for having a higher risk for T1D. TGFβ1 concentration in FDRs1, FDRs0, IRS, and CR, was lower than controls (p<0.001). IRS has a higher TGFβ1 concentration than CR (p<0.001).

**Conclusions:**

Stage 1, a higher risk for T1D, is characterized by decreases in CD25high T cells and TGFβ1, partially reflecting impaired T regulatory response, implying that changes of this T cells subset might be a risk marker for T1D. FDRs, irrespective of risk for T1D and T1D patients irrespective of state, had depletion of TGFβ1, suggesting the association of TGFβ1 could have potential with familiar risk and manifestation of T1D. Furthermore, the result suggested that the clinical course of overt T1D might be modulated on the TGFβ1 level.

## Abstract (sr)

**Uvod:**

Prethodne studije su pokazale važnu ulogu poremećaja nivoa T ćelijskih subsetova u različitim stadijumima razvoja tipa 1 dijabetesa (T1D), dok su podaci vezani za CD25high T ćelije i transformišući faktor rasta b1 (transforming growth factor-TGFb1), koji parcijalno reflektuju T regulatorni odgovor, i dalje kontroverzni. Analizirali smo nivo (a) CD25high T ćelija (b) TGFb1 kod 17 prvih rođaka pacijenata sa T1D u stadijumu 1 (first-degree relatives of patients with T1D - FDRs1) sa povećanim rizikom za T1D, (GADA+, IA-2+); 34 FDRs u stadijumu 0 (FDRs0) (GADA, IA-2); 24 pacijenta sa novootkrivenim T1D u insulin-zavisnom stanju (insulin requiring state-IRS); 10 pacijenata u kliničkoj remisiji (CR); 18 zdravih kontrola (CTR).

**Metode:**

Procenat CD4+CD25high T ćelija je analiziran metodom dvobojne imunofluorescencije i protočne citometrije, TGFb1 ELISA, GADA i IA-2 RIA metodom.

**Rezultati:**

Procenat CD25high T ćelija u FDRs1 je niži u poređenju sa kontrolama, FDRs0, IRS i CR (p<0,001). Procenat CD25high T ćelija od 1,19%, sa verovatnoćom 0,667, prediktuje povećan rizik za T1D. Koncentracija TGFb1 u FDRs1, FDRs0, i oba stanja u T1D, IRS i CR, je niža u poređenju sa kontrolama (p<0,001).

**Zaključak:**

Stadijum 1, povišen rizik za ispoljavanje T1D, karakterišu smanjenja u procentu CD25high T ćelija i koncentraciji TGFb1, delimično reflektujući oštećen imunoregulatorni odgovor, što bi mogao biti marker rizika za T1D. FDRs sa i bez rizika za T1D i pacijenti sa T1D bez obzira na stanje, su imali snižen TGFb1, što bi moglo da sugeriše potencijalnu udruženost TGFb1 sa familijarnim rizikom i ispoljavanjem T1D. Takođe, rezultati sugerišu da bi klinički tok T1D mogao biti potencijalno moduliran na nivou TGFb1.

## Introduction

Previous research indicates that defects in the number and function of CD4^+^ T cell subsets might be the risk marker for Type 1 diabetes (T1D) [1] [2] [3]. Simultaneously, the autoantibodies to pancreatic islet antigens are well-validated predictors of risk as well as diagnostic tools for T1D [4]. However, the association between the CD4^+^CD25^high^T cells subset, associated with T regulatory (T reg) response, and different T1D stages has not yet been fully elucidated [5] [6] [7] [8].

In that context, the course of T1D was defined through stages [9]. Stage 0 includes first-degree relatives (FDRs) of patients with T1D, who have 10–20 times higher relative risk of T1D compared to the general population, without islet autoantibodies and with normal glucose tolerance. Stage 1 is defined by two or more islet autoantibodies and euglycemia, stage 2 with multiple islet autoantibodies and dysglycaemia, and stage 3 is clinically manifested T1D [9] [10].

In that context, data regarding CD4^+^CD25^+^ T cells in different stages of T1D remains controversial, suggesting decreased, increased, or similar percentages compared to controls [5] [11] [12] [13] [14].

It is suggested that regulatory CD4^+^CD25^+^ T cells exert their suppressive effects due to the highest levels of CD25 expression within CD4^+^CD25^high^ subset [6] [15]. Precisely high expression of CD25 strongly attracts interleukin 2 (IL-2), leaving diabetogenic T cells without IL-2, which is necessary for their further development [6] [16] [17] [18]. Moreover, suppression can also occur via cell-to-cell-contact-dependent [19], as well as contact-independent mechanisms, by secretion of anti-inflammatory cytokines, transforming growth factor- β1 (TGF-β1), IL-10, IL-35, and other suppressive soluble factors [5] [12] [13]. TGF-β is a cytokine that inhibits immune responses, suppresses the functions of diabetogenic, proinflammatory Th1 cells, and promotes the generation of T reg cells [20]. However, the investigations focusing on TGF-β1 levels in different stages during the T1D course are limited.

Therefore, this study aimed to analyze the changes in (a) the percentage of CD4^+^CD25^high^ T cells’ subset and (b) TGFβ1 levels in peripheral blood in nondiabetic FDRs previously allocated in subgroups according to the stage in T1D development and patients with recent onset T1D (R-T1D).

## Materials and methods

### Subjects

In this study, we included 51 FDRs of the patients with T1D, 24 patients with R-T1D in insulin-requiring state (IRS), 10 patients with T1D in clinical remission (CR), and 18 healthy unrelated controls (CTR). The clinical characteristics of the investigated subjects were previously described [21]. Briefly, FDRs of patients with T1D were siblings and/or parents aged up to 45 years. We allocated them to 2 sub-groups: 17 FDRs were diagnosed in stage 1 (FDRs1) and were positive for the presence of glutamic acid decarboxylase (GADA) and tyrosine phosphatase insulinoma antigen-2 (IA-2A), higher risk for T1D developing, while 34 FDRs were diagnosed in stage 0 (FDRs0), negative for both autoantibodies, with lower risk for T1D developing.

The diagnosis of T1D was established according to the criteria set out by the Expert Committee of the American Diabetes Association [22] and confirmed by the presence of GADA and/or IA-2A. We included patients with T1D within 3 months of diagnosis in IRS and in the state of CR.

IRS in patients with R-T1D was defined as a necessity for insulin therapy to maintain euglycemia [23]. The patients were treated with standard basal-bolus injection insulin therapy (4 doses of insulin per day). Basal insulin was intermediate-acting human insulin given before bedtime to provide control of fasting glycemia (Insulatard HM 100 Novo Nordisk). Bolus insulin was short-acting human insulin given before main meals to control the glycemic rise at meals and to correct hyperglycemia (Actrapid HM100 Novo Nordisk). CR was defined as optimal metabolic control without insulin lasting longer than 30 days [24]. The control subjects had normal glucose tolerance, which was confirmed during a 2-hour 75-g oral glucose tolerance test (OGTT). There was no history of T1D in the family, and there was an absence of GADA and IA-2A [21].

Exclusion criteria for this investigation were: all subjects with acute or chronic diseases that could interfere with glucose homeostasis (with infective, allergic, and autoimmune diseases 6 months before blood samples being taken or who used immunomodulatory drugs at least 3 months before investigation).

### Research design

The study protocol was previously described [21]. In short, all FDRs were tested for the presence of GADA and IA-2A two times during the year. All FDRs and controls had normal glucose tolerance, verified by using a 2h OGTT. For patients with R-T1D, blood samples were collected in the morning, in fasting and euglycemic condition, within 3 months of diagnosis and initiation of basal-bolus insulin therapy after patients were admitted to the Clinic for Endocrinology, Diabetes and Metabolic Diseases University Clinical Center of Serbia, tertiary medical care level. The patients with overt T1D were evaluated in 2 different states during the clinical course, at the clinical onset of T1D in IRS and/or in the state of CR.

The metabolic investigations and detection of autoantibodies were performed in the Clinic for Endocrinology, Diabetes, and Metabolic Diseases after subjects gave informed consent to participate in the study, according to the Helsinki Declaration. Whole blood staining for flow cytometry analysis was conducted in the Institute for Medical and Clinical Biochemistry Faculty of Medicine University of Belgrade. The Institutional Review Board approved the study (Ethic Committee of Faculty of Medicine University of Belgrade, Decision number 1600/I-21, July 3, 2006).

### Detection of GADA and IA-2

Blood samples were obtained by standard venipuncture in BD vacutainer CAT early in the morning. We measured GADA and IA-2 using the radioimmunoassay method, due to the manufacturer’s instruction (CIS Bio International, Gif Sur Yvette, France), in duplicate. The inter-assay coefficients of variation (CV) were 4.9%, 7%, and 3.3, 5.3%, for the GADA and IA2, respectively, and the intra-assay CV were 3.6%, 3.7%, and 6.4 and 15.1, for all assays, respectively. Positive values were higher than 1 U/mL [21].

### Detection of glucose tolerance status

A 2-hour oral glucose tolerance test with 75 g of glucose was performed on each subject early in the morning, after 8h of fasting. Glucose stimulation was performed by orally ingesting glucose as a 50% solution for 3 minutes. Venous blood samples for determining the level of plasma glycemia were taken by venipuncture in BD vacutainers CAT in basal conditions immediately before glucose stimulation (0. minute) and after stimulation in the 30^th^, 60^th^, 90^th^ and 120^th^ minutes of the test [25]. The glycemia level was detected using the enzymatic method on Cobass 6000 (Roche Cobas 6000 Chemistry Analyzer (Roche Diagnostics Corporation, Indianapolis, IN 46250) with Roche, GLUC reagent kit (800 tests) Roche Diagnostics, Indianapolis, IN).

### Immunofluorescence staining and flow cytometry analysis

**Figure 1:**
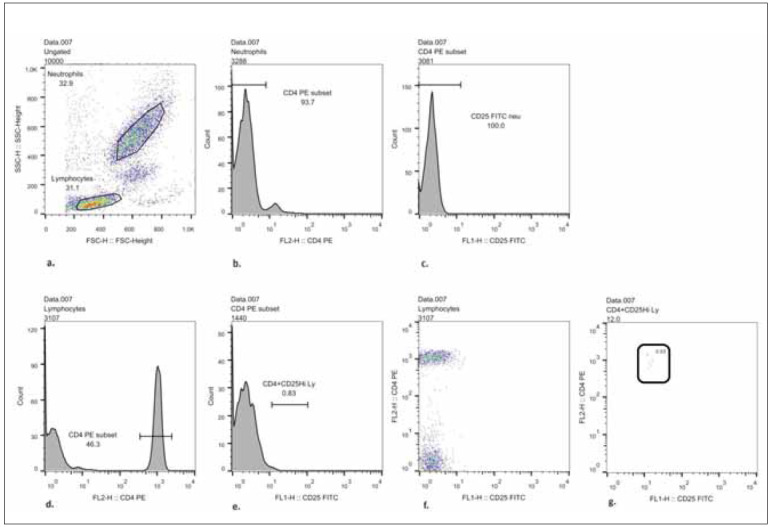
Representative histograms and dot plots of CD25 high cells in circulating CD4+ T cell population demonstrating gating strategy. Lymphocytes’ and neutrophils’ gates are created, as shown in Figure 1a. Figure 1b represents PE-fluorescence in neutrophils whereas Figure 1c represents PE-fluorescence in lymphocyte population CD4+ lymphocytes are marked as CD4-PE subset. The calculated GeoMean channel from Figure 1b, increased by 15 Robust SD of FITC fluorescence in Neutrophils, was used to define a threshold for CD25 high cells in the CD4+cell population (Figured). Figure 1e demonstrates no CD25high cells in the neutrophil population. Figure 1f shows FITC fluorescence in the lymphocyte region, whereas Figure 1g shows the percentage of CD4+CD25 high cells in the lymphocyte region.

The blood samples were obtained by venipuncture in the morning, into heparinized vacutainers (BD), and then treated with the procedure described earlier [21]. 100 microL of whole blood was incubated with saturating quantities of the appropriate anti-CD4 PE and anti-CD25 FITC-conjugated monoclonal antibodies for 30’ in the dark at room temperature. Isotype-matched FITC- and PE-conjugated irrelevant monoclonal antibodies were used as negative controls. After staining, erythrocyte lysis was performed using FACS Lysing solution (BD), and the cells were subsequently washed twice in PBS. Cells were immediately acquired (1–3×10^4^ leucocytes acquired per test) with FACSCalibur flow cytometer (BD Biosciences, San Diego, CA, USA) [21]. We have analyzed FCS files using FlowJo vX software (trial version). Forward and side scatter dot plots were used to define the lymphocyte and granulocyte populations. CD4^+^ T cells were defined as CD4^high^ population within the lymphocyte region. CD4^+^CD25^high^ positive cells were defined in each sample as CD4^+^ cells with higher FITC fluorescence than the threshold determined by channel of Geometric Mean of FITC fluorescence in Neutrophils (CD25 negative cell population) increased by 15 Robust SD of FITC fluorescence in Neutrophils. The number of CD4^+^CD25^high^ cells was expressed as a percentage of the CD4^+^CD25^high^ cells in the CD4^+^cell population (see gating strategy, Figure 1a-g*). (The following Ab’s from BD PharMingen (San Diego, CA, USA) were used in our study: IgG1 (679.1Mc7-FITC), IgG2 (U7.27-PE), fluorescein isothiocyanate (FITC)-conjugated monoclonal anti-human CD25 (clone M-A251) and phycoerythrin-labeled anti-CD4 (clone SK3).

Figure 1a, b, c, d, e, f, g. Representative histograms and dot plots of CD25 ^high^ cells in circulating CD4^+^ T cell population demonstrating gating strategy. Lymphocytes’ and neutrophils’ gates are created, as shown in Figure 1a. Figure 1b represents PE-fluorescence in neutrophils whereas Figure 1c represents PE-fluorescence in lymphocyte population CD4^+^ lymphocytes are marked as CD4-PE subset. The calculated GeoMean channel from Figure 1b, increased by 15 Robust SD of FITC fluorescence in Neutrophils, was used to define the threshold for CD25 ^high^ cells in the CD4^+^cell population (Figure 1d). Figure 1e demonstrates no CD25^high^ cells in the neutrophil population. Figure 1f shows FITC-fluorescence in the lymphocyte region, whereas Figure 1g shows the percentage of CD4^+^CD25 ^high^ cells in the lymphocyte region.

### Detection of TGF-β1 level in peripheral blood

TGF-β1 was measured by a solid-phase ELISA (Genzyme, Cambridge, MA), and the procedures are previously described in patients with multiple sclerosis [26]. In that context, serum samples, run in duplicate, under the manufacturer’s instructions, were acidified with HCl for 1 h to release the biologically active form of TGF-β1 the inactive complex formed from the noncovalent association of a mature TGF-β dimer and a second dimer (latency-associated protein). Intra- and interassay coefficients of variation were 4.1% and 7.2%, respectively.

### Statistics

Data are presented as mean ± SD. Data were tested for normal distribution using Kolmogorov–Smirnov test. Kolmogorov-Smirnov test showed the absence of Normal distributions (all p-values were under 0.001). The Man-Whitney test was conducted to detect statistically significant differences between observed categories. Two-tailed p-values less than 0.05 were considered significant. Data were analyzed using the Statistical Package for the Social Sciences (SPSS) software (Advanced Statistics, version 26.0), Chicago, IL.

## Results

### Clinical characteristics

The overview of clinical characteristics of subjects involved in the investigation is shown in Table 1. All participants were nonobese adults, matched according to age and gender.

**Table 1:** Characteristics of first-degree relatives of patients with T1D (FDRs) at stage 0, (FDRs0) and stage 1 (FDRs1) for development of T1D, patients with recent-onset T1D (R-T1D) in insulin-requiring state (IRS), patients in clinical remission of T1D (CR) and healthy controls (CTR) included in CD4+ T cells and TGFβ1 analysis in the peripheral blood. Body mass index (BMI), HbA1c (glycated hemoglobin), FPG (fasting plasma glucose)

	FDRs1	FDRs0	RT1D-IRS	CR	CTR
Number	17	34	24	10	18
Gender (m/f)	4/13	18/16	15/9	4/6	2/16
Age (yrs.)	29.82±8.83	26.44±6.09	26.43±6.02	26.22±5.06	28.18±7.21
BMI (kg/m^2^)	23.71±2.66	22.69±3.72	21.40±3.47	22.12±2.71	22.00±4.21
Duration of T1D<br>(months)	/	/	2.30±0.52	9.20±2.68	/
HbA1c (%)	/	/	9.70±0.86	7.06±0.42	/
FPG (mmol/L)	4.84±1.06	4.22±1.31	5.2±0.8	5.8±0.4	4.50±1.20

### Analysis of the level of CD4^+^CD25^high^ T cells in peripheral blood

**Figure 2:**
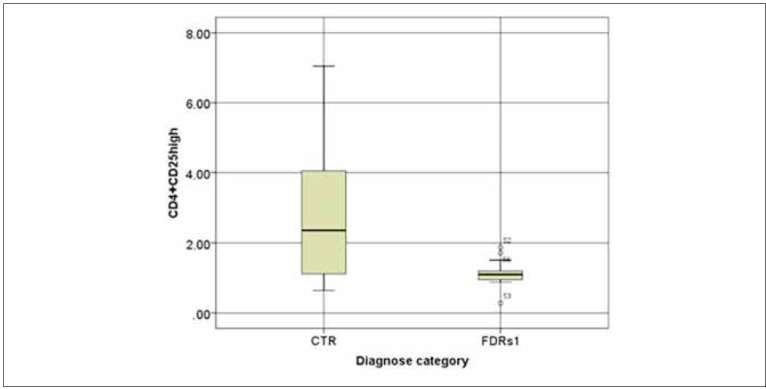
The percentage of CD4+CD25^high^ in peripheral blood: comparing first-degree relatives in stage 1 (FDRs1) and healthy controls (CTR). The percentage of CD4+CD25^high^ T cells of the total number of peripheral blood lymphocytes was determined by immunofluorescence and flow cytofluorometry. The horizontal lines represent the median, and the box plots include the 25th and 75th percentile and error bars of the 10th and 90th percentiles. Outliers are marked. The Man-Whitney test was conducted to detect statistically significant differences between observed categories. FDRs1 vs CTR p<0.01.

The percentage of CD4^+^CD25^high^ T cells in FDRs1 was significantly lower compared to control subjects, FDRs0 as well as patients with R-T1D in IRS and CR (FDRs1: 1.1 (0.925–1.2) vs CTR: 2.36(1.09–4.09) (p<0.01), FDRs1: 1.1 (0.925–1.2) vs FDRs0 2.31 (1.71–3.29) (p<0.001), FDRs1: 1.1 (0.925–1.2) vs R-T1D IRS: 2.18 (1.61–2.93) (p<0.001) FDRs1: 1.1 (0.925–1.2) vs CR: 2.33 (1.99–2.56) % (<0.001) (Figure 2, Table 2).

**Table 2:** Descriptive analysis of CD25high and TGFβ1 levels through 5 investigated groups (first-degree relatives of patients with T1D (FDRs) at stage 0, (FDRs0) and stage 1 (FDRs1) for development of T1D, patients with recent-onset T1D (R-T1D) in insulin-requiring state (IRS), patients in clinical remission of T1D (CR) and healthy controls (CTR)). Kolmogorov-Smirnov test showed the absence of normal distributions (all p values were under 0.001). The Man-Whitney test was conducted to detect statistically significant differences between observed categories. *** – p-value <0.001; ** – p-value<0.01; * – p-value<0.05

Indicators	The median (25th and 75th percentile) are shown					MW
	CTR	FDRs1	FDRs0	R-T1D IRS	CR	
CD25^high^<br>cells within CD4^+<br>^lymphocytes (%)	2.36<br>(1.09–4.09)	1.1<br>(0.925–1.2)	2.31<br>(1.71–3.29)	2.18<br>(1.61–2.93)	2.33<br>(1.99–2.56)	IRS>FDRs1 ***<br>CR>FDRs1 ***<br>FDRs0>FDRs1 ***<br>CTR>FDRs1 **
TGFβ 1 (pg/mL)	10015.00<br>(9737.25–10682.50)	4982.00<br>(4199.00–5570.5)	5323.5<br>(5178.75–5483.50)	5495.00<br>(5380–5682.50)	4000.00<br>(3900–4190)	CTR>IRS ***<br>CTR>CR ***<br>CTR>FDRs1 ***<br>CTR > FDRs0***<br>IRS>CR ***<br>IRS>FDRs1 **<br>FDRs1>CR *<br>FDRs0 vs FDRs1 NS<br>FDRs0 vs IRS NS

Simultaneously, the percentage of CD4^+^CD25^high^ T cells in patients with R-T1D in IRS and CR was lower compared to control subjects but without statistical significance (p=NS), as well as between patients in IRS and CR (p=NS) (Table 2).

**Figure 3:**
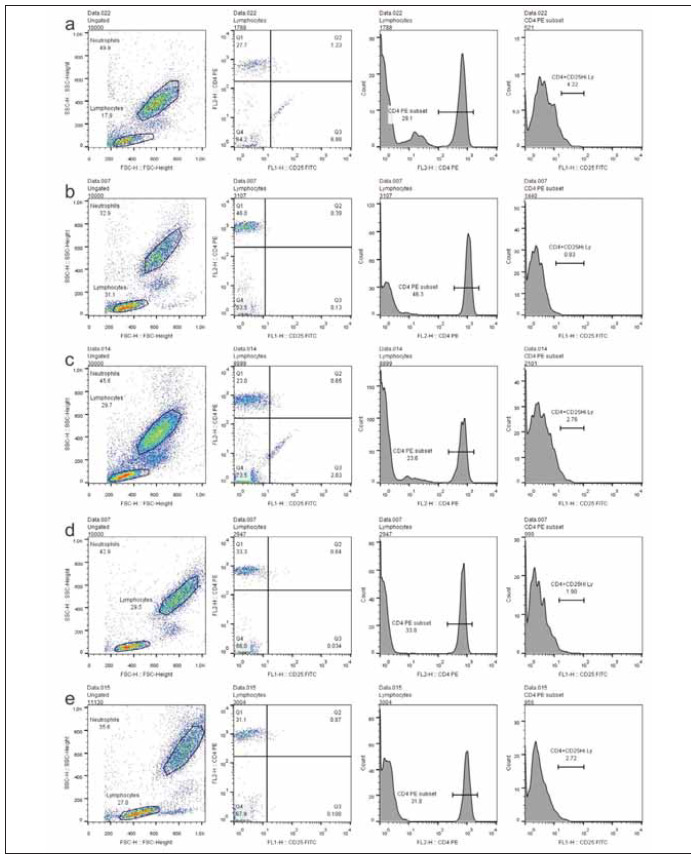
Dot plots and histograms of representative specimens collected from 5 investigated groups of subjects: healthy controls (CTR) (a), first-degree relatives of patients with T1D at stage 1 – FDRs1 (b), first-degree relatives of patients with T1D at stage 0 – FDRs0 (c), patients with recent-onset T1D in insulin-requiring state (IRS) (d) and patients in clinical remission of T1D (CR) (e). Respective diagrams (left to right) represent: FSc/SSc dot plot of peripheral blood lymphocytes (far left panel), events within lymphocyte region presented on CD25/CD4 dot plot (middle left panel), histogram of CD4 fluorescence distribution within the lymphocyte region (middle right panel) and histogram of CD25 fluorescence distribution within the CD4+ lymphocyte population (far right panel).

The data from representative specimens collected from each group of subjects are presented in Figure 3.

### Analysis of the level of TGFβ1 in the peripheral blood

**Figure 4:**
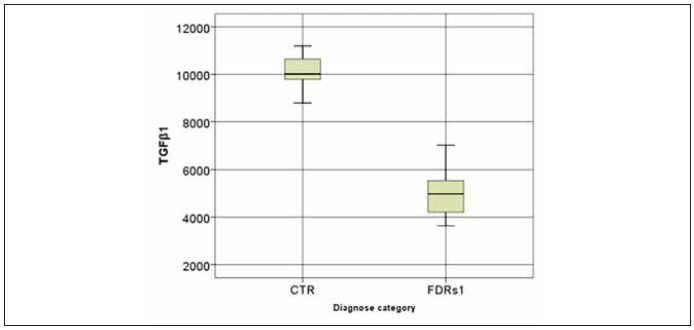
The concentration of TGFβ1 in peripheral blood (pg/mL): comparing first-degree relatives in stage 1 (FDRs1) and healthy controls (CTR). The horizontal lines represent the median, and the box plots include the 25th and 75th percentile and error bars of the 10th and 90th percentiles. Outliers are marked. The Man-Whitney test was conducted to detect statistically significant differences between observed categories. FDRs1 vs CTR p<0.001.

We found that the concentration of TGFβ1 in FDRs1 was significantly lower compared to control subjects and comparable to FDRs0 (FDRs1: 4982.00 (4199.00–5570.5) vs CTR: 10015.00 (9737.25–10682.50) (p<0.001), FDRs1: 4982.00 (4199.00–5570.5) vs FDRs0: 5323.5 (5178.75–5483.50) (p=NS) (pg/mL)) (Figure 4, Table 2). At the same time, the concentration of TGFβ1 in different stages of manifested T1D, IRS, and CR, respectively, was significantly lower compared to control subjects, too (R-T1D IRS: 5495.00 (5380–5682.50) vs CTR: 10015.00 (9737.25–10682.50) (p<0.001), CR: 4000.00 (3900–4190) vs CTR: 10015.00 (9737.25–10682.50) (p<0.001) (pg/mL)) (Table 2).

Additionally, patients with R-T1D in IRS have a significantly higher concentration of TGFβ1 compared to the patients in CR as well as to FDRs1, and comparable to FDRs0 (R-T1D IRS: 5495.00 (5380–5682.50) vs CR: 4000.00 (3900–4190) (p<0.001), R-T1D IRS: 5495.00 (5380–5682.50) vs FDRs1: 4982.00 (4199.00–5570.5) (p<0.01) R-T1D IRS: 5495.00 (5380–5682.50) vs FDRs0: 5323.5 (5178.75–5483.50) (p=NS)) (pg/mL)) (Table 2).

### Defining the percentage of CD4^+^CD25^high^ T cells that separates FDRs at higher risk for developing T1D and control subjects

**Figure 5:**
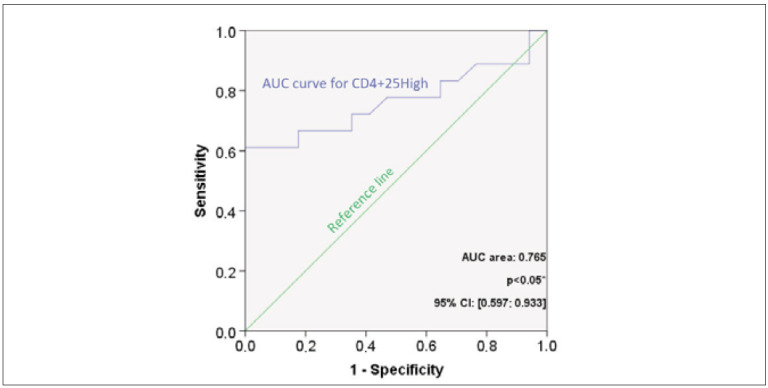
ROC curve analysis of CD25^high^ scores.

**Figure 6:**
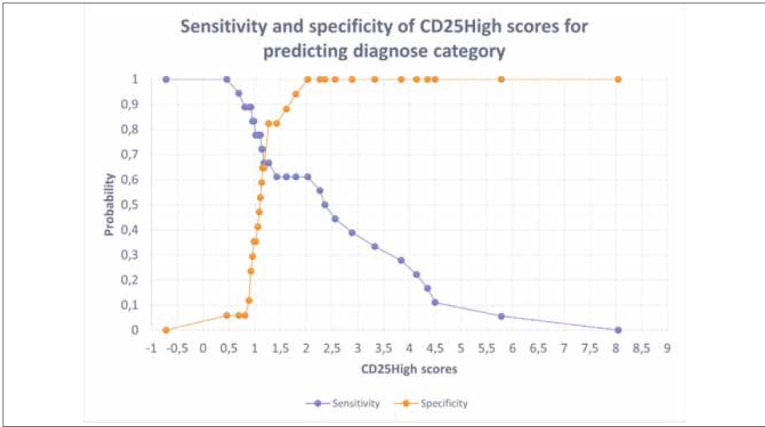
Overview of sensitivity and specificity analysis of CD25^high^ scores with the cut-off value for CD25^high^ = 1.19%, with probability 0.667.

We also determined a cut-off value, i.e. the percentage of CD4^+^CD25^high^ T cells in the peripheral blood, at which it is possible to separate the FDRs1 and the control subjects. In that context, a binary logistic regression was conducted to determine specificity and sensitivity cut-off values for predicting diagnostic categories, i.e., participating in diagnostic category healthy controls and FDRs1. Firstly, the created model showed an AUC value of 0.765, close to the generally acceptable cut-off value for successful tests (0.8). Furthermore, the model has been tested for its sensitivity and specificity scores, where positive classes were associated with predicting diagnostic category FDRs1. Considering the results pinpointed in Figure 5, 4.1. and Figure 6, CD25^high^ scores might be perceived as valuable predictors of diagnostic category where the cut-off value for sensitivity and specificity is 1.19% of CD25^high^ score (probability of 0.667).

## Discussion

We have demonstrated that the stage 1, state of increased risk for T1D development in nondiabetic FDRs of patients with T1D, is associated with the lowest CD25highT cells and diminished TGFβ1, partially reflecting impaired T regulatory response. This finding implies that the lowest level of that CD4^+^ T cells subset might be a risk marker for T1D development. However, all FDRs, irrespective of risk for T1D, and all T1D patients, irrespective of state, had depletion of TGFb1, an anti-inflammatory cytokine, potentially suggesting the association of TGFβ1 with familiar risk and manifestation of T1D. Furthermore, the results suggested that the clinical course of manifested T1D might be potentially modulated on the level of TGFβ1.

Our results are in line with the results of studies that found a decreased number of immune regulatory T cells defined as CD4+CD25+ T cells in high-risk FDRs [11] [12].

In that context, the first study examined CD4^+^CD25^+^ T cells in only 2 pediatric FDRs with positive GAD or/and IA2, detected lower levels of these T cells compared to controls, and similar to R-T1D and long-standing T1D, although all of them were not adequately matched according to the age [11]. Moreover, interesting data were published in a prospective study on FDRs in the pediatric population, where 2 subpopulations, immune regulatory T cells defined as CD4^+^CD25^+^ T cells and natural killer T cells, were determined [12]. FDRs with higher genetic risk for T1D had fewer CD4^+^CD25^+^ T cells.

Interestingly, opposite results published in the study analyzed again pediatric FDRs with autoantibodies, which had higher levels of immune regulatory T cells defined as CD4^+^CD25^+^HLADR^-^ and CD4^+^ CD25^+^CD69^-^, implying intensification of immune regulatory response during preclinical stages in T1D [5]. However, like in previous studies, subjects were not matched adequately according to age, considering the important effect of age on the frequency of CD25^+^FOXP3^-^ T cells, which corresponds with CD4^+^CD25^low^ T cells [14]. In addition, some studies reported the absence of alteration in CD4^+^CD25^+^ CD127^low^FOXP3^+^T cells in autoantibody-positive at risk children [27].

Finally, the lowest percentage of CD25^high^ T cells in FDRs1, with a higher risk for T1D, potentially reflects their escape into inflamed pancreatic islets in the preclinical phase of T1D. Moreover, the level of CD25^high^ T cells might be the marker of early auto-immunity and early defect in immunoregulatory response. Furthermore, we detected a cut-off point for sensitivity and specificity, the value 1.19% of CD25^high^ T cells (probability of 0.667), that might be realized as a valuable predictor of diagnostic category for higher risk for T1D.

When we analyzed the percentage of CD25^high^ T cells in patients with R-T1D in IRS and CR, we showed a lower percentage of CD25^high^ in both stages of manifested T1D than in controls, although without statistical significance. These results agree with most studies published till now, analyzing similar subpopulations [7] [14] [28] [29].

In that context, [28] mini meta-analysis of 4 studies that analyzed the role of regulatory CD4^+^ CD25^+^ T cells in T1D reported predominantly similar values as in control subjects [7] [14] [29] except in one study showing lower values in T1D patients [11].

Moreover, the absence of alteration of the number of wider subpopulation of CD4^+^ T cells was accompanied by no difference in the level of iRNA for CD25 and TGFβ in T cells between T1D patients and controls, which is partially in line with our results [30] [31] [32].

However, a recently finished study reported decreased Helios, a novel marker for T reg detection in T1D patients, suggesting multiple maturation defects in the T reg subpopulation [33]. In contrast, another study revealed more CD4^+^CD25^+^CD127^low^FOXP3^+^ T regs in children with T1D than in controls [27].

Additionally, we showed that patients in CR had a slight increase in the level of CD25^high^ T cells, which is in agreement with the published results [8] [34] [35] [36].

In that sense, a previous study revealed insulin induced Foxp3 protein expression in CD25^high^ T cells in children with R-T1D, suggesting that treatment with an autoantigen, i.e. insulin, induces T reg activation and may contribute to the induction of CR [8]. However, the transient nature of CR suggests impaired suppressor function in CD4^+^CD25^+^ T-cells in patients with T1D [34].

In that context, children with a higher percentage of CD25^high^ T cells subset had a remission state more frequently, although this difference diminished after 2 years. However, unlike us, CR was defined as total insulin daily dose <0.5IU/kg and HbA1c<7% [35].

Simultaneously, it was recently reported that patients with the highest percentage of CD4^+^ CD25^+^CD127^hi^ cells at the beginning of the disease had the longest duration of CR, comprising a mix of Th1- and Th2-type cells, with the dominance of anti-inflammatory Th2-type cells [36].

On the other hand, the level of circulating cytokines is thought to be a marker of cellular immunity. In this context, we analyzed the level of TGFβ1, a cytokine that partially reflects CD25^high^ T cell activity and T reg response.

Our FDRs1 and FDRs0 had similarly decreased TGFβ1 concentration than healthy controls. This finding implied that TGFβ1 level alone might not be associated with markers of humoral autoimmunity but is related to familiar risk for T1D. At the same time, patients with T1D had severely diminished concentration of TGFβ1. Interestingly, patients in IRS had higher TGFβ1 concentration than in CR, suggesting an intensification of regulatory anti-inflammatory response as an attempt to adapt and block the proinflammatory milieu in the early phase of manifested T1D. Later on, during CR, a decrease in TGFβ1 might be the consequence of further functional exhaustion of CD25^high^ T cells subset with anti-inflammatory features. Having that in mind, TGFβ1 concentration might potentially modulate the clinical course of overt T1D.

Limited data in this area are available. In line with our data, patients with R-T1D showed higher levels of Th3 cytokines TGF-β and IL-10 than nondiabetic high-risk children [11] [37]. Moreover, it was shown that patients with R-T1D have lower mRNA levels for TGF-β in unstimulated peripheral mononuclear cells compared to healthy controls [38] [39]. Finally, increasing the TGF-β and T regs levels represents a form of immunotherapy in T1D [40] [41].

Our study has some limitations, such as using only a CD25 marker on CD4+T cells to reflect the Treg response partially. Since we did not use other markers, such as FOXP3, we detected cells that express the CD25 antigen with the strongest intensity, covering most of the immune regulatory subpopulation [6] [42]. Moreover, we did not analyze in vitro T reg functionality after stimulation, but the level of TGF-β1, a cytokine that might be produced by T reg and several other lymphoid and non-lymphoid cells in peripheral blood. Since this is a cross-sectional study, individual genetic differences between patients cannot be adequately controlled as in longitudinal studies, nor can causality among parameters be established. Additionally, we included a rather moderate number of subjects.

On the other hand, unlike most studies that address this topic, we included well-defined homogeneous groups, exclusively FDRs in stage 0 (lower risk) and stage 1 (higher risk for T1D), as well as recent onset T1D in insulin-requiring state and complete CR without insulin therapy, to exclude the influence of insulin therapy and duration of the disease. When tested, all patients were in euglycemic conditions, bearing in mind that hyperglycemia can influence the quantity of CD4^+^ T cells. Finally, the majority of the previous studies were done in children, where auto - immunity has a dominant role in pathogenesis, while our results were obtained in an age-matched population of adults.

In conclusion, we have demonstrated that the different stages in adult T1D are accompanied by changes in CD25^high^ lymphocytes and TGFβ1 level, which incompletely describe the impaired immune regulatory response. In that context, increased risk for T1D in FDRs1 is associated with diminishing this cell subset and potentially might be a risk marker for T1D. However, FDRs, irrespective of risk for T1D, and T1D patients, irrespective of state, had depletion of TGFβ1, suggesting the association of TGFβ1 could have potential with familiar risk and manifestation of T1D. Moreover, the clinical course of overt T1D might be potentially modulated on the level of TGFβ1. Further studies are needed to include a larger number of persons at risk for T1D, exploring immune regulatory cells, especially in the adult population, which steadily increases the number of patients.

## Dodatak

### List of abbreviations

type 1 diabetes, T1D;

first-degree relatives, FDR);

T regulatory cells, T regs;

glutamate decarboxylase antibodies, GADA;

tyrosine phosphatase insulinoma antigen-2 antibodies, IA-2;

transforming growth factor β1, TGFβ1;

insulin-requiring state, IRS;

clinical remission, CR.

### Acknowledgements

This work was funded by Project 451-03-66/2024-03/200110 from the Ministry of Science, Technological Development and Innovations of the Republic of Serbia.

### Conflict of interest statement

All the authors declare that they have no conflict of interest in this work.
